# Clinical characteristics and poor predictors of anti-NXP2 antibody-associated Chinese JDM children

**DOI:** 10.1186/s12969-020-00492-z

**Published:** 2021-01-06

**Authors:** Xinning Wang, Yuchuan Ding, Zhixuan Zhou, Jun Hou, Yingjie Xu, Jianguo Li

**Affiliations:** Department of Rheumatology and Immunology, The Affiliated Children’s Hospital, Capital Institute of Pediatrics, 2 Yabao Road, Chaoyang District, Beijing, 100020 China

**Keywords:** Juvenile dermatomyositis, Anti-nuclear matrix protein 2, Chinese

## Abstract

**Background:**

Juvenile dermatomyositis (JDM) is a rare and sometimes fatal disease in children. The anti-NXP2 antibody is one of the most common antibodies and muscle ischaemia associated with NXP2 autoantibodies was a severe subtype of JDM. Further information is needed regarding clinical characteristics and factors associated with poor prognosis. But there are no reports about clinical characteristics and high risk factor of poor prognosis. For the first time, we introduced the clinical characteristics and poor predictors of anti-NXP2 antibody-associated juvenile dermatomyositis in Chinese children.

**Methods:**

Twenty-six patients with anti-NXP2 antibody-related JDM from 85 JDM Chinese patients were diagnosed from January 2016 to November 2019. Logistic regression was used to analyze the risk factors for refractory cases and mortality.

**Results:**

The ratio of male to female was 1:1.9. The median age of onset was 4.5 (1–13) years. Twenty-four cases (92.3%) had rash and muscle weakness. Treatments included glucocorticoids, immunosuppressive agents, biological agents (7 cases), plasma exchange, Janus kinase inhibitor (7 cases) and autologous stem cell transplant (1 case). Refractory JDM patients (11/26, 42.3%) were associated with edema, skin ulcer, muscle strength<=grade 3, CD4/CD8 ratio < 1.4 and ferritin > 200μg/ml. Among 6 cases (6/26, 23.1%) with severe gastrointestinal involvement, 5 cases died and 1 case survived after autologous stem cell transplant (ASCT). The risk factors for gastrointestinal involvement and mortality were edema, skin ulcer, severe muscle weakness (dysphagia/ hoarseness/ soft voice), BMI < 15 and ANA positive.

**Conclusions:**

Edema, skin ulcer and severe muscle weakness predicted refractory disease, GI involvement, and mortality in anti-NXP2 antibody-positive JDM of Chinese children. Decreased CD4/CD8 ratio and high ferritin related with refractory cases, and very low BMI and ANA (+) are probably, associated with gastrointestinal involvement and mortality.

**Trial registration:**

http://www.chictr.org.cn/showproj.aspx?proj=49846.

## Background

Juvenile dermatomyositis (JDM) is a pediatric-onset idiopathic inflammatory myopathy associated with autoimmune vasculopathy, mainly characterized by proximal muscle weakness and typical rash [1]. The incidence rate is (2 ~ 4/100,0000) [2]. China has a huge population of children, while there are few reports on the incidence and clinical features of JDM in Chinese children.

JDM is a highly heterogeneous disease, involving not only muscles and skin, but also other organs including cardiovascular, respiratory, and gastrointestinal systems and even cause considerable mortality [3]. In recent years, myositis-specific autoantibodies (MSAs) were found to be closely associated with distinct clinical features and prognosis of the DM sub-types. It is reported that anti-NXP2 antibody, might be one of the most common MSA groups in JDM which has been reported to have poor outcomes [4, 5]. Some case reports suggested that gastrointestinal involvement was a serious complication in anti-NXP2 antibody-related JDM [5, 6].

Further information is needed regarding features associated with refractory cases and risk factors for death of anti-NXP2 antibody-positive JDM. We present retrospective analysis of features, treatment and outcomes of anti-NXP2 antibody-related Chinese JDM cases.

## Patients and methods

### Patients

Chinese children diagnosed with anti-NXP2 autoantibody positive JDM at Capital Institute of Pediatrics (CIP) from January 2016 to November 2019 were included. Specifically, the inclusion criteria were: 1) age < 18 years old; 2) diagnosed as JDM based on the Bohan and Peter criteria for myositis [4]; 3) anti-NXP2 antibody positive. The exclusion criteria were 1): other diseases that cause weakness or rash (clear alternative diagnosis), or 2) unwillingness to enroll in the study. The age of disease onset was defined as the earliest age that the typical symptoms of JDM appeared. Duration before diagnosis indicates the time from onset to diagnosis. Refractory was defined as the ineffectiveness of glucocorticoids combined with more than two immunosuppressive agents, and (or) need more aggressive treatment like biological agents. Very low BMI was defined as BMI < 13. The first-line treatment is Glucocorticoid (GC) combining immunosuppressant. If the response to the treatment is not ideal, another immunosuppressant or biological agents will be added. In the study, all cases were initially treated with GC and immunosuppressive agents except one girl for her parents refused to use GC. This study was approved by Ethics Committee of CIP (KSSHERLL2017068).

### Laboratory examinations

We used immune dot blot to determine the presence of anti-NXP2 antibody in human serum (D-TEK, Belgium). Creatine kinase (CK) were tested by automated biochemical analyzer (Siemens, Germany). Ferritin was measured by quantitative chemiluminescence assay (Abbott Laboratories, US). Anti-nuclear antibodies (ANA) and anti-Ro-52 antibodies were measured by immunofluorescence assay (Euroimmun, US) and immunoblotting (Euroimmun, US) respectively. We assessed CD4/CD8 ratio by flow cytometry and immunofluorescence (Becton, Dickinson and Company, US).

### Statistical analyses

Characteristics of patients were compared with four kinds of outcomes. Indicators include age at onset, duration, BMI, muscle strength, childhood myositis assessment score (CMAS), CK, ferritin and CD4/CD8 ratio. Continuous variables are expressed as median and range (minimum-maximum) and categorical variables were expressed as frequency and percentage. Associations between the risk factors for poor outcomes were evaluated by univariate logistic regression analysis. Adjusted odds ratio (OR) and 95% confidence interval (95% CI) were calculated. In addition, follow-up time was compared using Wilcoxon’s signed rank test and each variable was evaluated by Spearman correlation test. *P* values < 0.05 (two-sided) were considered as statistically significant. All statistical analyses were performed with R 3.2.3 (http://www.r-project.org/).

## Results

### Patients

Of 85 patients with JDM, 26 were anti-NXP2 autoantibody positive. Among the 26 patients, 13 cases including 4 severe patients were transferred from other hospitals. Ages at onset of these patients ranged from 1 to 13 years with the median age of 4.5 years. Nine cases were found CMAS<=2. Baseline characteristics of patients are shown in Table 1. The demographic and clinical characteristics of the patients are presented in Table 2.

**Table 1:** Clinical characteristics of 26 JDM patients with anti-NXP2 autoantibody positive

ID	gender	Age*	Duration	BMI (kg/m^2^)	First symptom			CMAS	Dysphagia/ Hoarseness/ Lower Voice	Ulcer/ Edema	max CK (U/L)	SF (μg/ml)	ANA/ Anti-Ro-52	CD4/CD8 ratio	ILD	Gastro-intestinal	Treatment	Follow -up(m)	Clinical partial response at 3rd month	CMAS at the end of the follow-up
					Rash	Weakness	both													
1	M	8	1	18.1		Y		23	N	N/N	1794	67	N/N	2.01	N	N	1 + 2 + 0 + 0 + 0	41	N	52
2	M	1	42	15.6			Y	16	N	N/N	Nd	48	Y/N	1.54	N	N	1 + 2 + 0 + 0 + 0	49	N	45
3	M	8	1	19.5		Y		23	Y	N/N	3873	202	Y/N	0.83	N	N	1 + 1 + 0 + 0 + 0	47	Y	52
4	F	11	7	15.2	Y			25	N	N/N	Nd	97	N/N	2.25	N	N	1 + 4 + 0 + 0 + 0	60	Y	52
5	F	9	3	21.9			Y	47	Y	N/N	8138	120	N/N	1.68	N	N	1^*^ + 2 + I + 0 + 1	16	Y	47
6	F	4	6	24.6			Y	22	Y	N/N	535	132	N/N	2.03	N	N	1^*^ + 3 + I + 0 + 0	15	Y	52
7	F	4	6	15.7	Y			47	N	N/N	516	50	N/N	2.23	N	N	1^*^ + 1 + I + 2 + 1	14	N	47
8	M	3	12	15.7			Y	22	N	Y/N	Nd	141	N/N	1.52	N	N	1 + 0 + 0 + 0 + 0?	Nd	Nd	Nd
9	F	6	Nd	Nd			Y	23	N	N/N	757	Nd	N/N	2.18	N	N	1 + 2 + 0 + 0 + 0	14	Y	52
10	M	1	2	17.9			Y	4	Y	N/N	621	474	Y/N	2.07	N	N	1^*^ + 2 + I + 0 + 0	16	N	47
11	F	7	1	22.6			Y	28	N	N/N	922	14	N/N	1.7	N	N	1 + 3 + 0 + 0 + 0	51	N	52
12	F	2	1	14.1			Y	8	N	N/N	582	193	N/N	2.08	Y	N	1^*^ + 1 + I + 0 + 0	13	Y	52
13	F	6	1	18.2	Y			0	Y	N/N	3327	178	N/N	2.3	Y	N	1^*^ + 3 + I + 0 + 0	18	Y	52
14	F	5	1	14.9	Y			8	N	N/N	3392	279	Y/Y	1.28	N	N	1^*^ + 2 + I + 0 + 0	22	Y	49
15	F	4	3	16.4	Y			0	N	N/N	1010	227	N/Y	1.7	N	N	1^*^ + 3 + I + 0 + 0	34	Y	47
16	F	6	1	16.2		Y		0	Y	N/Y	15,140	783	N/Y	1.93	N	N	1^*^ + 2 + I + 0 + 1	16	Y	40
17	F	13	1	29.1			Y	6	Y	Y/N	1156	239	Y/N	1.25	Y	N	1^*^ + 3 + I + 1 + 1	31	Y	47
18	F	1	4	16.6			Y	25	N	N/N	221	362	N/N	2.33	N	N	1^*^ + 3 + I + 1 + 0	29	N	45
19	M	1	3	17.5	Y			0	Y	Y/Y	1096	347	N/Y	1.16	Y	N	1^*^ + 3 + I + 0 + 1	28	N	18
20	M	1	3	15.3			Y	0	Y	Y/Y	2000	185	Y/Y	0.81	N	N	1^*^ + 4 + I + 1 + 1	12	N	33
21	F	1	3	13.9	Y			2	Y	Y/Y	1250	323	Y/N	ND	N	Y	1^*^ + 5 + I + 1 + 1 + ASCT	12	N	27
22	F	5	1	13.3		Y		0	Y	Y/Y	10,064	584	Y/N	0.75	Nd	Y	1^*^ + 3 + I + 0 + 0	0.1	Y	/
23	F	3	2	13.6			Y	5	Y	Y/Y	Nd	Nd	Y/N	Nd	N	Y	1^*^ + 3 + I + 0 + 0	43	N	/
24	M	4	1	16.1	Y			5	Y	Y/Y	118	132	Y/N	1.14	Y	Y	1^*^ + 5 + I + 1 + 0	12	N	/
25	F	6	1	11.3			Y	2	Y	Y/Y	6457	416	Y/Y	0.68	Y	Y	1^*^ + 3 + I + 0 + 0	4	N	/
26	F	9	1.5	12.8		Y		2	Y	Y/Y	3178	1566	Y/Y	1.74	Y	Y	1^*^ + 1 + I + 1 + 0	1	N	/

*Y* yes, *N* no, *Nd* no data, *Age* age at onset, *Duration* time from onset to diagnosis, *BMI* body mass index, *CMAS* childhood myositis assessment score, *CK* creatine kinase, *SF* serum ferritin, *ANA* antinuclear antibody, *ILD* interstitial lung disease, *Treatment* MP + immunosuppressive agent(s) + IVIG + biological agents (not including JAK inhibitors) + JAK inhibitor, 1^*^ MP + high dose MP pulse, *I* IVIG, *ASCT* autologous stem cell transplantation; Immunosuppressive agents: methotrexate (MTX), cyclophosphamide (CTX), cyclosporine (CsA), azathioprine, tacrolimus, mycophenolate mofetil (MMF), hydroxychloroquine, thalidomide, etc.

**Table 2:** Demographic and clinical data of 26 patients with anti-NXP2 autoantibody positive JDM

Baseline characteristics	n (%) or Median (range)
**Diagnosis**	
JDM	26(100%)
**Gender**	
Male	9(34.6%)
Female	17(65.4%)
**Age at onset (y)**	4.5(1–13)
**Duration from onset to diagnosis (m)**	2.0(1.0–42.0)
**First symptom**	
Rash	8(30.8%)
Muscle weakness	5(19.2%)
Rash & muscle weakness	13(50%)
**Proximal muscle strength**	
< = grade 3	18(69.2%)
> grade 3	8(30.8%)
**CMAS**	7(0–47)
**dysphagia/hoarseness/soft voice**	15(57.7%)
**Other signs**	
Cutaneous ulceration	10(38.5%)
Periorbital edema	5(19.2%)
Calcification	3(11.5%)
**Other organ systems**	
Arthritis	8(30.8%)
Interstitial lung disease	7(26.9%)
Gastrointestinal involvement	6(23.1%)
**Laboratory testing**	
CK (ng/ml)	1203(118–15,140)
Serum ferritin (ng/ml)	198(14–1566)
ANA (+) only	8(30.8%)
Anti-Ro52 antibody (+) only	3(11.5%)
ANA (+) & Anti-Ro52 antibody (+)	4(15.4%)
**Other examination**	
MRI: myositis	26
EMG: myogenic damage	18(81.8%)
HRCT: interstitial lung disease	7(28%)
**Treatment**^**a**^	
GC + IA^≤2^ ± (IVIG)	8(30.8%)
GC + IA^>2^ ± (IVIG)	8(30.8%)
GC + IA^≤2^ + IVIG+BA	4(15.4%)
GC + IA^> 2^ + IVIG+BA	6(23.1%)
**Follow-up(m)**	27.5(1.0–106.0)
**Refractory JDM**	11(42.3%)
**Death**	5(19.2%)

*JDM* juvenile dermatomyositis, *JPM* juvenile polymyositis, *CMAS* childhood myositis assessment score, *CK* creatine kinase, normal range: 50–220 U/L; serum ferritin normal range: 15–200 ng/ml, *ANA* antinuclear antibody, *MRI* magnetic resonance imaging, *EMG* electromyography, *HRCT* high-resolution computed tomography

^a^GC: glucocorticoids including methylprednisolone and prednisolone; IA: immunosuppressive agents; IA^≤2^: two or less than 2 types of immunosuppressive agents; IA^>2^: more than 2 types of immunosuppressive agents; IVIG: intravenous immunoglobulin; BA: biological agents, including monoclonal antibodies and JAK inhibitors like tofacitinib. m: months for follow-up.

### Clinical assessment

Initial CK was recorded in 22 patients (the results of 4 cases transferred from other hospitals were not available. Two cases (2/22, 9.1%) were within normal limits,, twelve cases (12/22, 54.5%) were > 1-10x upper limit of normal (ULN) and eight cases were > 10x ULN. ANA and anti-Ro-52 antibody status are shown in Table 2. All of the positive ANA titers were 1:100. Results of imaging examinations including Magnetic resonance imaging (MRI) and High-resolution CT (HRCT) were also showed in Table 2. Pulmonary function was characterized by mild obstructive ventilation dysfunction, decreased small airway function, and 3 cases had mildly increased residual volume.

### Treatment and prognosis

Treatment started promptly upon diagnosis. Regimens were showed in Table 1. (GC) was the first-line therapy, except one case started with biological agent because parents refused to use GC. If GC plus two immunosuppressive agents failed, more immunosuppressive agents or/and biological agents were be given. One case received autologous stem cell transplantation (ASCT) for severity and poor response to medicine. Tofacitinib (Janus-kinase inhibitors, JAKi) or Ruxolitinib was used in 7 cases. Two were treated with JAKi due to intractable rash at 4 months and 1 year respectively; four were treated with JAKi because of the aggressive nature of the disease or no remission with GC and immunosuppressive agents; and one was treated with JAKi after ASCT for refractory rash. Twenty-five patients were followed up for 27.5 (1.0–106.0) months (one case was lost for follow-up). Twenty patients improved and five died of gastrointestinal perforation or related complications; One patient with gastrointestinal involvement (intestinal edema) survived after ASCT.

### Statistical analyses results

#### Comparison between refractory and non-refractory groups

**Fig. 1:**
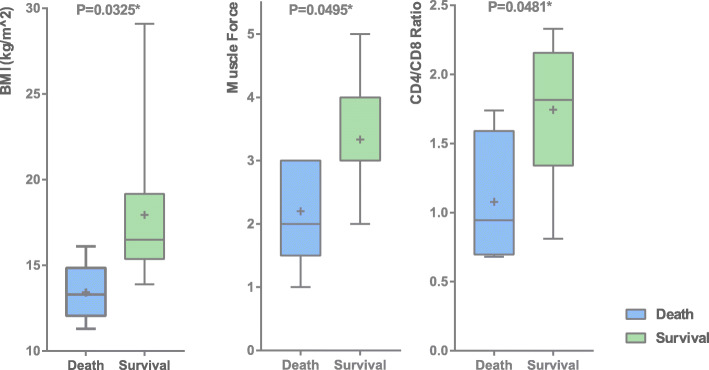
The difference of BMI, muscle strength and CD4/CD8 ratio between survival and death

Among 26 patients, 11 cases (11/26, 42.3%) were refractory and 15 were non-refractory. The differences of muscle strength, CMAS, SF and CD4/CD8 ratio between refractory and non-refractory group are shown in Fig. 1. There was no significant difference in BMI and CK between the two groups (Supplement 1). Furthermore, we analyzed the predictors of refractory JDM by univariate logistic regression (Table 3). Edema (*P* < 0.0001), skin ulcers (*P* = 0.0003), severe muscle weakness (dysphagia/hoarseness/soft voice, *P* = 0.0089; muscle strength<= grade 3, *P* = 0.0041), CD4 / CD8 ratio < 1.4 (*P* = 0.0255) and ferritin > 200 ng / ml (*P* = 0.0361) were considerably associated with refractory JDM. ANA positive might be correlated with refractory JDM, but the difference was not significant (*P* = 0.0521). BMI and Ro-52 are not associated with refractory cases (*P* > 0.05).

**Table 3:** Univariate logistic regression of refractory and non-refractory groups of anti-NXP2 autoantibody positive JDM

		Outcome ratio (%)	N	β	OR	95%CI	***P***-values
Gender	male	33.33	26	0.5534	1.739	0.257 ~ 14.451	0.8037
	female	47.06					
BMI (kg/m^2^)	> = 15	33.33	25	1.5396	4.663	0.558 ~ 62.967	0.2032
	< 15	71.43					
Muscle strength	> 3	0	26	2.6654	14.373	2.461~ > 999.999	0.0041*
	<=3	61.11					
Edema	No	11.76	25	3.926	50.705	8.086~ > 999.999	<.0001*
	Yes	100					
Skin ulcer	No	12.5	26	3.8559	47.27	3.869~ > 999.999	0.0003*
	Yes	90					
Dysphagia/hoarseness/soft voice	No	9.09	26	2.8634	17.521	1.691 ~ 952.094	0.0089*
	Yes	66.67					
Symptom	No	10	26	2.5977	13.433	1.308 ~ 721.398	0.0219*
	Yes	62.5					
ILD	No	27.78	25	1.7855	5.963	0.698 ~ 82.561	0.1237
	Yes	71.43					
ANA (+)	No	21.43	26	1.9024	6.702	0.987 ~ 61.614	0.0521
	Yes	66.67					
Anti-Ro-52 (+)	No	31.58	26	1.6178	5.042	0.609 ~ 67.675	0.1696
	Yes	71.43					
CD4/CD8 ratio	> = 1.4	18.75	24	2.4221	11.269	1.26 ~ 171.48	0.0255*
	< 1.4	75					
Ferritin (ng/ml)	<=200	16.67	24	2.1876	8.914	1.115 ~ 123.379	0.0361*
	> 200	66.67					

*Outcome ratio* the ratio of outcomes in different condition, *Symptom* at least one of edema, skin ulcer or Dysphagia/Hoarseness/Lower voice, *BMI* body mass index, *ILD* interstitial lung disease, *ANA* antinuclear antibody; *: significantly statistic difference, *P* < 0.05

### Comparison between survival and death groups

**Fig. 2:**
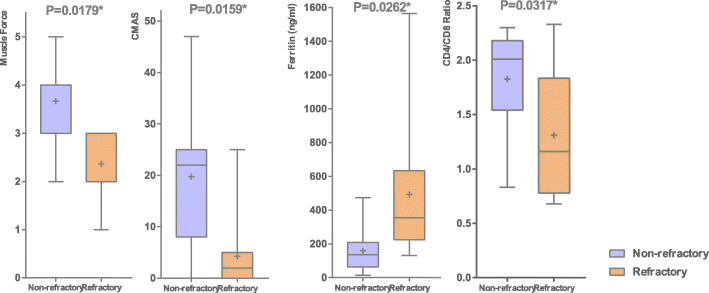
The difference of muscle strength, CMAS, ferritin and CD4/CD8 ratio between refractory JDM and non-refractory JDM

BMI (*P* = 0.0325), muscle strength (*P* = 0.0495) and CD4 / CD8 ratio (*P* = 0.0481) were significantly different between death and survival groups (Fig. 2). There was no significant difference in CMAS scores, CK and ferritin (Supplement 2). Univariate logistic regression analysis found that BMI < 15 (*P* = 0.012) and ANA positive (*P* = 0.0245) were highly correlated with mortality (Table 4). Regarding the cause of death, 5 cases of children died of gastrointestinal involvement. The variables significantly associated with gastrointestinal involvement generally overlapped with the variables significantly associated with death. (Supplement 3).

**Table 4:** Univariate logistic regression of survival and death groups of anti-NXP2 autoantibody positive JDM

		Outcome ratio (%)	N	Β	OR	95%CI	*P* value
Gender	Male	11.11	26	0.8695	2.386	0.188 ~ 136.137	0.8394
	Female	23.53					
BMI (kg/m2)	> = 15	5.56	25	2.9305	18.737	1.328~ > 999.999	0.0245*
	< 15	50					
Muscle strength	> 3	0	26	1.2982	3.663	0.585~ > 999.999	0.1303
	<=3	27.78					
Edema	No	0	25	2.8369	17.062	2.562~ > 999.999	0.0055*
	Yes	50					
Skin ulcer	No	0	26	2.8358	17.045	2.722~ > 999.999	0.0038*
	Yes	50					
dysphagia/hoarseness/soft Voice	No	0	26	1.8624	6.439	1.044~ > 999.999	0.0457*
	Yes	33.33					
Symptom	No	0	26	1.6794	5.362	0.867~ > 999.999	0.0664
	Yes	31.25					
ILD	No	5.56	25	2.4074	11.105	0.696 ~ 707.076	0.1051
	Yes	42.86					
ANA (+)	No	0	26	2.4202	11.248	1.819~ > 999.999	0.012*
	Yes	41.67					
Anti-Ro-52 (+)	No	15.79	26	0.7253	2.065	0.136 ~ 24.28	0.8215
	Yes	28.57					
CD4/CD8 ratio	> = 1.4	6.25	24	2.0877	8.066	0.517 ~ 502.932	0.1818
	< 1.4	37.5					

*Outcome ratio* the ratio of outcomes in different condition, *Symptom* at least one of edema, skin ulcer or Dysphagia/Hoarseness/Lower voice, *BMI* body mass index, *ILD* interstitial lung disease, *ANA* antinuclear antibody, *: significantly statistic difference, *P* < 0.05

As to treatment of JDM, during the follow up, 6 patients’ CMAS were significantly increased. Nine severe cases including those with gastrointestinal involvement received biological agents treatment, such as rituximab and infliximab. JAKi was used in 7 cases because of no response to conventional therapy. All 7 cases showed good response on JAKi. A severe case, with skin ulcer, edema, muscle weakness and gastrointestinal involvement, received salvage treatment with ASCT. After ASCT, her situation was significantly improved; 2 years later, she went to kindergarten and no longer needed any medicine.

## Discussion

JDM is a rare idiopathic inflammatory myopathy. Studies showed that more than 2 thirds of patients developed a chronic course and 4.1% of patients died [7, 8], patients have to taken drugs for years and some depends on wheelchair, which seriously affectsed patients’ quality of life and social participation. MSA is potentially useful biomarker for it is associated with different clinical phenotypes [9, 10]. In children with JDM, anti-MDA5, anti-TIF-1γ and anti-NXP2 are the most common MSAs [11–15]. The reports from UK and Argentina showed anti-NXP2 antibody were detected in 23 and 25% respectively of patients with JDM [16, 17]. In our study, anti-NXP2 seemed to be the most common antibody (30.6%). The ratio of anti-NXP2 antibody in JDM was higher than that in western countries [4, 17, 18], which may be because the severe patients from all over the country concentrated to our department. The present study reported the characteristics and high risk factors of poor response to the treatment and death of anti-NXP2 antibody-related JDM in Chinese.

Twenty-six patients with anti-NXP2 antibody out of 85 JDM patients were involved. The female to male ratio was 1.9:1, which was different from reports of adult DM patients that anti-NXP2-antibodies were predominantly found in men [19, 20]. The median age at onset is 4.5 years, which was younger than the average age of whole JDM cases [21].

As to the onset symptoms, muscle weakness and skin rash were the most common clinical manifestations of the JDM patients in this cohort (92.4%). The skin lesions in this study included rashes (96.2%), skin ulcer (38.5%), edema (19.2%) and calcification (11.5%). Calcification was reported to be highly associated with the anti-NXP2 antibodies [5],while only 11.5% of patients showed calcification in our cohort, which might be related to active treatment due to the severe weakness [22]. In the cohort, 96.2% of patients manifested muscle weakness during the course of disease. Serious weakness (strength<=grade 3) was found in 69.2% of patients. In the 15 patients with a CMAS < 10, 9 cases were found CMAS<=2 and all manifested symptoms of palatal, laryngeal, and pharyngeal muscles group including dysphagia/hoarseness/soft voice. Magnetic resonance imaging (MRI) is preferred imaging modality for its noninvasive and no radiation. It is helpful in detecting sensitively monitor disease activity and muscle damage [23]. MRI showed muscle involvement in all 26 patients including those without muscle weakness. There was a girl, whose MRI revealed significant abnormalities but she had not muscle weakness at all, suggesting the importance of MRI in evaluating muscle involvement. Electromyography (EMG) showed myogenic damage in 69.2% of cases, suggesting it was not as sensitive as MRI [23].

The level of serum muscle enzymes plays an important role in the diagnosis of JDM. At the onset of the disease, 9.1% cases were normal, 54.5% of cases varied from 1 to 10 times the normal levels and 36.4% of cases were higher than 10 times the normal levels. It was interesting that the CK level were almost normal in some patients who died of severe JDM; While in some cases who were sensitive to therapy or with mild symptoms, CK was significantly increased (> 10,000 U/L). The phenomenon suggested that the level of CK was not associated with disease severity. As reported in the previous study, the markers that are currently used in clinical practice, AST, ALT, LDH, aldolase and in particular CK activity, do not correlate as well with disease activity in JDM as in DM [24]. Ueki M found in their studies [11], that 50% of cases in the study were found with mildly elevated level of ALT, but whether it was liver dysfunction or not remains to be validated.

Previous assessment in America noted uncommon lung involvement in anti-NXP2 autoantibody JDM. A study of Caucasians reported that none of those cases with anti-NXP2 antibody JDM had any lung involvement [3, 25]. However, in the study, HRCT revealed 26.9% of patients had mild ILD, while the corresponding pulmonary manifestations such as dyspnea and cough were not obvious. ILD quickly disappeared after treatment, which was not same as anti-MDA5 antibody-related JDM.

Of the 26 cases, 5 died of gastrointestinal perforation, which accounted for the vast majority of 6 deaths (6/85), suggesting that anti-NXP2 antibody related complications accounted for majority of death in Chinese children with JDM. Among the death group, 4 of 5 patients were female. Statistical analysis showed significant differences of BMI, edema, skin ulcer, dysphagia/hoarseness/soft voice, ANA (+) was found between death group and survival group as well as in groups with and without gastrointestinal involvement.

Among the 5 deaths, very low BMI (< 13) could be seen in 4 children. Univariate logistic regression analysis found BMI < 15 (*P* = 0.012) was highly correlated with death. The very low BMI might be a result of chronic gastrointestinal before perforation [26]. There are limitations of the study: Firstly, our center is a large referral center and likely to receive more severe and more refractory cases which may lead to a biased population. Secondly, the present study is a retrospective approach and based on only 1 medical center. What’s more, the length of follow-up for assessment of death is limited and lack of function status assessment and disease course (monocyclic, polycyclic, remission). Multicenter and large sample study needs to be carried out to verify the findings.

## Conclusions

We observed that patients presenting with edema, skin ulcer and dysphagia/hoarseness/soft voice are more associated with requiring more aggressive therapy and more associated with mortality. Decreased CD4/CD8 ratio and high ferritin are associated with refractory cases. Further more, Low BMI and positive ANA were associated with gastrointestinal involvement and mortality. Further prospective multicenter study is needed to verify these findings.

## Supplementary Information

**Additional file 1: Supplement 1**. Characteristics of Refractory and Non-refractory.

**Additional file 2: Supplement 2.** Characteristics of Death and Survival and Anti-NXP2 antibody-positive JDM With and Without Gastrointestinal Involvement.

**Additional file 3: Supplement 3**. univariate logistic regression results in anti-NXP2 antibody-positive JDM with and without gastrointestinal involvement.

## Data Availability

All data generated or analyzed during this study are included in this published article and its supplementary information files.

## Abbreviations

NXP2: Nuclear matrix protein 2
TIF-1γ: Transcriptional intermediary factor 1γ
MDA5: Me lanoma differentiation associated gene
JDM: Juvenile dermatomyositis
JPM: Juvenile polymyositis
IIM: Idiopathic inflammatory myopathies
MSA: Myositis-specific autoantibody
ASCT: Autologous stem cell transplant
JAKi: Janus kinase inhibitor
IVIG: Intravenous immunoglobulin
BMI: Body mass index
ANA: Anti-nuclear antibody
HRCT: High-resolution computed tomography
IVIG: Intravenous immunoglobulin
MRI: Magnetic resonance imaging
ILD: Interstitial lung disease
EMG: Electromyography
CMAS: Childhood myositis assessment score
CK: Creatine kinase
