# Supplementary material for: Clinical characteristics and poor predictors of anti-NXP2 antibody-associated Chinese JDM children
Source: Pediatr Rheumatol Online J. 2021 Jan 6;19:6. doi: 10.1186/s12969-020-00492-z (PMC7788734; doi:10.1186/s12969-020-00492-z)
Supplement: Supplementary file 1 — Additional file 1: Supplement 1. Characteristics of Refractory and Non-refractory. [file 12969_2020_492_MOESM1_ESM.docx]

Supplement 1. Characteristics of Refractory and Non-refractory

|  | Refractory  n=11 | Non-refractory  n=15 | β | P value | OR | 95%CI |
| --- | --- | --- | --- | --- | --- | --- |
| Age at onset (y) | 4(1-13) | 5(1-11) | -0.0696 | 0.5785 | 0.933 | 0.73~1.192 |
| Duration (m) | 1.5(1-4) | 2.5(1-42) | -0.2988 | 0.1993 | 0.742 | 0.47~1.171 |
| BMI (kg/m2) | 15.3(11.3-29.1) | 17.15(14.1-24.6) | -0.146 | 0.2469 | 0.864 | 0.675~1.106 |
| Muscle force | 2(1-3) | 4(2-5) | -2.461 | 0.0179* | 0.085 | 0.011~0.655 |
| CMAS | 2(0-25) | 22(0-47) | -0.1389 | 0.0159* | 0.87 | 0.777~0.974 |
| CK (U/L) | 1625(118-15140) | 966(516-8138) | 0.000157 | 0.254 | 1 | 1~1 |
| SF (ng/ml) | 354.5(132-1566) | 136.5(14-474) | 0.01 | 0.0262* | 1.01 | 1.001~1.019 |
| CD4/CD8 ratio | 1.16(0.68-2.33) | 2.01(0.83-2.3) | -2.1432 | 0.0317* | 0.117 | 0.017~0.828 |

Duration: time from onset to diagnosis; BMI: body mass index; CMAS: childhood myositis assessment score; CK: creatine kinase; SF: serum ferritin.

*: significantly statistic difference, P<0.05
