# Supplementary material for: Clinical characteristics and poor predictors of anti-NXP2 antibody-associated Chinese JDM children
Source: Pediatr Rheumatol Online J. 2021 Jan 6;19:6. doi: 10.1186/s12969-020-00492-z (PMC7788734; doi:10.1186/s12969-020-00492-z)
Supplement: Supplementary file 2 — Additional file 2: Supplement 2. Characteristics of Death and Survival and Anti-NXP2 antibody-positive JDM With and Without Gastrointestinal Involvement. [file 12969_2020_492_MOESM2_ESM.docx]

Supplement 2. Characteristics of Death and Survival and Anti-NXP2 antibody-positive JDM With and Without Gastrointestinal Involvement

|  | Death  n=5 | Survival  n=21 | β | P value | OR | 95%CI | Gastrointestinal involvement  n=6 | No-gastrointestinal involvement  n=20 | β | P value | OR | 95%CI |
| --- | --- | --- | --- | --- | --- | --- | --- | --- | --- | --- | --- | --- |
| Age at onset (y) | 5(3-9) | 4(1-13) | 0.0501 | 0.7379 | 1.051 | 0.784~1.41 | 4.5(1-9) | 4.5(1-13) | -0.037 | 0.8004 | 0.964 | 0.723~1.284 |
| Duration (m) | 1(1-2) | 3(1-42) | -0.9065 | 0.1957 | 0.404 | 0.102~1.595 | 1.25(1-3) | 3(1-42) | -0.4892 | 0.2366 | 0.613 | 0.273~1.379 |
| BMI (kg/m2) | 13.3(11.3-16.1) | 16.5(13.9-29.1) | -1.3389 | 0.0325* | 0.262 | 0.077~0.894 | 13.45(11.3-16.1) | 16.6(14.1-29.1) | -1.6225 | 0.0248* | 0.197 | 0.048~0.814 |
| Muscle force | 2(2-3) | 3(2-5) | -1.6156 | 0.0495* | 0.199 | 0.04~0.996 | 2(1-3) | 3(2-5) | -1.973 | 0.0278* | 0.139 | 0.024~0.807 |
| CMAS | 2(0-5) | 16(0-47) | -0.1528 | 0.1364 | 0.858 | 0.702~1.049 | 2(0-5) | 19(0-47) | -0.1681 | 0.1 | 0.845 | 0.692~1.033 |
| CK (U/L) | 4817.5(118-10064) | 1126(221-15140) | 0.00014 | 0.277 | 1 | 1~1 | 3178(118-10064) | 1096(221-15140) | 0.000099 | 0.4234 | 1 | 1~1 |
| SF (ng/ml) | 500(132-1566) | 189(14-783) | 0.00416 | 0.1022 | 1.004 | 0.999~1.009 | 416(132-1566) | 185(14-783) | 0.00405 | 0.1039 | 1.004 | 0.999~1.009 |
| CD4/cd8 ratio | 0.945(0.68-1.74) | 1.815(0.81-2.33) | -2.838 | 0.0481* | 0.059 | 0.004~0.976 | 0.945(0.68-1.74) | 1.815(0.81-2.33) | -2.838 | 0.0481* | 0.059 | 0.004~0.976 |

Duration: time from onset to diagnosis; BMI: body mass index; CMAS: childhood myositis assessment score; CK: creatine kinase; SF: serum ferritin.

*: significantly statistic difference, P<0.05
