# Supplementary material for: Clinical characteristics and poor predictors of anti-NXP2 antibody-associated Chinese JDM children
Source: Pediatr Rheumatol Online J. 2021 Jan 6;19:6. doi: 10.1186/s12969-020-00492-z (PMC7788734; doi:10.1186/s12969-020-00492-z)
Supplement: Supplementary file 3 — Additional file 3: Supplement 3. univariate logistic regression results in anti-NXP2 antibody-positive JDM with and without gastrointestinal involvement. [file 12969_2020_492_MOESM3_ESM.docx]

Supplement 3. univariate logistic regression results in anti-NXP2 antibody-positive JDM with and without gastrointestinal involvement

|  |  | Outcome ratio (%) | n | β | OR | 95%CI | P value |
| --- | --- | --- | --- | --- | --- | --- | --- |
| Gender | Male | 11.11 | 26 | 1.1629 | 3.199 | 0.276~177.031 | 0.5913 |
|  | Female | 29.41 |  |  |  |  |  |
| BMI (kg/m2) | >=15 | 5.56 | 25 | 3.4758 | 32.324 | 2.304~>999.999 | 0.0043* |
|  | <15 | 71.43 |  |  |  |  |  |
| Muscle force | >3 | 0 | 26 | 1.5629 | 4.773 | -0.2423~>999.999 | 0.0806 |
|  | <=3 | 33.33 |  |  |  |  |  |
| Edema | No | 0 | 25 | 3.2889 | 26.813 | 4.151~>999.999 | 0.0011* |
|  | Yes | 62.5 |  |  |  |  |  |
| Skin ulcer | No | 0 | 26 | 3.2038 | 24.627 | 4.003~>999.999 | 0.0009* |
|  | Yes | 50 |  |  |  |  |  |
| Dysphagia/Hoarseness/Lower Voice | No | 0 | 26 | 2.1479 | 8.567 | 1.429~>999.999 | 0.0217* |
|  | Yes | 33.33 |  |  |  |  |  |
| Symptom | No | 0 | 26 | 1.6794 | 5.362 | 0.867~>999.999 | 0.0664 |
|  | Yes | 31.25 |  |  |  |  |  |
| ILD | No | 5.56 | 25 | 1.7008 | 5.478 | 0.466~87.656 | 0.2261 |
|  | Yes | 42.86 |  |  |  |  |  |
| ANA(+) | No | 0 | 26 | 2.7422 | 15.52 | 2.572~>999.999 | 0.004* |
|  | Yes | 50 |  |  |  |  |  |
| Anti-Ro-52 (+) | No | 21.05 | 26 | 0.389 | 1.476 | 0.104~14.716 | 1 |
|  | Yes | 28.57 |  |  |  |  |  |
| CD4/CD8 ratio | >=1.4 | 6.25 | 24 | 2.0877 | 8.066 | 0.517~502.932 | 0.1818 |
|  | <1.4 | 37.5 |  |  |  |  |  |

Outcome ratio: the ratio of outcomes in different condition; Symptom: at least one of edema, skin ulcer or dysphagia/hoarseness/lower voice; BMI: body mass index; ILD: interstitial lung disease; ANA: antinuclear antibody; SF: serum ferritin.

*: significantly statistic difference, P<0.05
